# IoT Sensing for Reality-Enhanced Serious Games, a Fuel-Efficient Drive Use Case

**DOI:** 10.3390/s21103559

**Published:** 2021-05-20

**Authors:** Rana Massoud, Riccardo Berta, Stefan Poslad, Alessandro De Gloria, Francesco Bellotti

**Affiliations:** 1 Department of Electrical, Electronics and Telecommunication Engineering and Naval Architecture (DITEN), University of Genova, 16145 Genova, Italy; rana.massoud@elios.unige.it (R.M.); alessandro.degloria@unige.it (A.D.G.); franz@elios.unige.it (F.B.); 2 IoT Laboratory, Queen Mary University of London, London E1 4NS, UK; stefan.poslad@qmul.ac.uk

**Keywords:** Internet of Things (IoT), serious game (SG), reality-enhanced serious games (RESGs), eco-driving, fuel consumption, on-board diagnostic-II (OBD-II), machine learning (ML)

## Abstract

Internet of Things technologies are spurring new types of instructional games, namely reality-enhanced serious games (RESGs), that support training directly in the field. This paper investigates a key feature of RESGs, i.e., user performance evaluation using real data, and studies an application of RESGs for promoting fuel-efficient driving, using fuel consumption as an indicator of driver performance. In particular, we propose a reference model for supporting a novel smart sensing dataflow involving the combination of two modules, based on machine learning, to be employed in RESGs in parallel and in real-time. The first module concerns quantitative performance assessment, while the second one targets verbal recommendation. For the assessment module, we compared the performance of three well-established machine learning algorithms: support vector regression, random forest and artificial neural networks. The experiments show that random forest achieves a slightly better performance assessment correlation than the others but requires a higher inference time. The instant recommendation module, implemented using fuzzy logic, triggers advice when inefficient driving patterns are detected. The dataflow has been tested with data from the enviroCar public dataset, exploiting on board diagnostic II (OBD II) standard vehicular interface information. The data covers various driving environments and vehicle models, which makes the system robust for real-world conditions. The results show the feasibility and effectiveness of the proposed approach, attaining a high estimation correlation (R^2^ = 0.99, with random forest) and punctual verbal feedback to the driver. An important word of caution concerns users’ privacy, as the modules rely on sensitive personal data, and provide information that by no means should be misused.

## 1. Introduction

Reality-enhanced serious games (RESGs) is an emerging game genre, spurred also by the spread of the Internet of Things (IoT) [1], in which a player’s progress is not only due to his or her digital gaming ability, but also tied to measurements from the field [2,3]. In this way, a user’s performance in the field becomes an immediate gaming factor [2].

In this area, the REAL [4] framework has been designed to support the use of IoT sensor data in different instances of serious games (SGs), that are games with a different goal (e.g., instructional or training) rather than pure entertainment [5]. REAL was tested with a suite of smartphone-based RESGs for mobility, including: an arcade “Passenger Game”, with the player’s energy being related to the quality of driving; a “Driver Game” with a minimal user interface; a classic “Competition“ which compared eco-drive scores of drivers along a common path; and a more complicated “Snake and ladders” game that exploits parking-related events. Exploiting the REAL bridge helps SG developers to focus on designing the desired game logic via plugging in different field sensors seamlessly. Reference [6] shows the core integration architecture and a serious game use case of a gamification framework that leverages the potential of the IoT paradigm to closely link actions, decisions and events happening in real-life with in-game educational progress and gaming technologies.

However, little attention has been spent in the literature, so far, on smart modules for processing of the field sensor signals to be used in RESGs. The games in [4] used smartphone and vehicular sensors to estimate driving quality through simple expert rules, while recent advances in machine learning (ML) have provided significant opportunities for extracting high-level information from raw sources [7]. Our paper thus intends to advance the state of the art of RESG design by (i) investigating the feasibility of a reference model to provide effective information from the field to RESG players and (ii) analyzing whether ML algorithms can support a proper implementation. The model should be usable in different game implementations, as listed, for instance, in the previous paragraph.

As a use case, we focus on automotive driver’s fuel efficiency. Fuel consumption (FC) due to transportation accounts for a significant share of the total fuel oil output [8]. The driver’s style (e.g., aggressive, defensive, calm) also plays a significant role there. Reference [9] reports a 30% difference in FC due to driving styles on a light-duty vehicle in the urban driving cycle. The same percentage is reported by [10] in energy consumption for an electric vehicle. Real-time information from automotive sensors can be useful to improve driver behavior, which in turn is expected to improve fuel-efficiency [11]. Therefore, feedback to the driver through eco-driving support has been recommended [12,13]. Drivers should be provided with precise and understandable information, as advice could be misinterpreted [14]. We argue that these make the case significant, even at high levels of automation, as it is important that drivers are at least aware of the impact of driving styles on fuel consumption level [15]. Hence, the research hypotheses that we seek to substantiate in this research are: Fuel Consumption can be accurately and instantaneously estimated based on data from selected standard sensors inbuilt in a car engine and via a standard interface to these sensors; Fuel consumption estimates can be used to advise and instantaneously offer guidance on drivers’ behavior to aid them to drive more fuel efficiently.

Given these two hypotheses and given the importance of the two aspects of quantitative user performance assessment and coaching, we propose a reference model that is pluggable into third party RESGs and that combines the following two smart sensing modules:

(1) Instant assessment. A RESG relies on a continuous flow of user behavior assessment measurements. This flow can be exploited by a game design more or less directly through a variety of mechanics, such as an ongoing score that might be compared in a hall of fame with peers, or for self-comparisons. Other possible examples of mechanics include the energy of a player’s avatar or power of an enemy weapon, activation of bonuses or maluses, etc. (e.g., [3]). Feedback to the driver may also include profile categorization (e.g., saver, normal, consumer, etc.—considering our FC use case) and a report of the recognized driving patterns.

(2) Instant recommendations. Besides the assessment, it is valuable to also provide users/players (e.g., drivers, in our use case) with guidance on how to improve (e.g., what actions could be taken to reduce inefficient driving patterns), provided through an adequate human-computer interaction [16]. To this end, the algorithms should provide verbal feedback that is easily understandable and applicable by drivers. 

**Figure 1:**
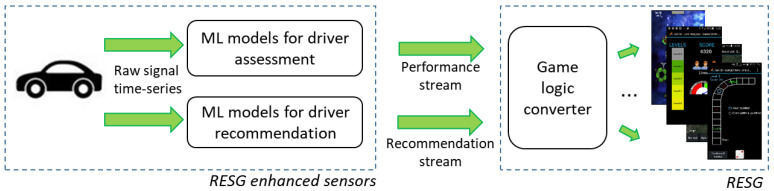
The driver assessment and recommendation modules process vehicular signals and provide information usable inside different types of games.

For both modules, the real-time response requirement is dictated by the driver’s reaction time, so it should be considered to be of the order of a hundredth of a second [17]. The overall targeted workflow is shown in Figure 1. The figure shows the RESG enhanced sensor model, including the two ML-based modules for instant assessment and instant recommendation. The modules process the raw vehicular signal time-series (e.g., speed, acceleration pedal position, steering wheel angle) data and output the performance and recommendation streams, respectively. The first one is continuous, while the second one contains information only when events are detected that deserve providing verbal feedback to the driver. For the sake of completeness, the figure also shows that these streams need to be processed by each RESG according to its own logic, as illustrated in [4]. For instance, a game may use the instant performance value as the energy factor for a hero (or to compute the energy of enemies) or accumulate it for the creation of bonuses. The focus of this paper is on the left part of the figure (i.e., on enhancing sensor data).

The remainder of this article is organized as follows: Section 2 reviews the literature; Section 3 presents the experimental settings; Section 4 explores algorithms for real-time FC estimation, while Section 5 focuses on instant driving recommendations; Section 6 analyzes a case study; while Section 7 discusses issues, conclusions and future work.

## 2. Literature Review

The practice of inserting real-world measurements into an instructional/training gameplay is well known in the area of serious games for energy. The EnergyLife game—which was designed to support the users’ actions and embeds contextualized feedback triggered by specific actions of the user, called ‘smart advice’—has been one of the first design attempts considering users’ own personal energy consumption behavior, tailoring feedback accordingly [18]. Reference [19] presents a field test of a professional quality social game about energy use in a virtual home. Smart meter data showed a significant decrease in electricity usage compared with 30-day periods before and after play. Reference [20] reviews the design and effectiveness of 10 games that aim to influence household energy consumption and presents a gamification approach in which real world activities are implemented in a game design. The same authors later argued that the Powersaver Game is effective in transferring energy conservation knowledge to users, which led to increased energy saving behavior on the long term [21]. A similar approach has been introduced in the health domain as well. For instance, [22] present a set of exergames that not only keeps track of the user’s vital state, but also directly integrates vital parameters into the gameplay.

The term Reality Enhanced Serious Games stems from these experiences and indicates a generalization to various domains. In the mobility domain, serious games directly fed by real-world inputs have been proposed both for private [4,23] and public [2] transportation. The spread of IoT technologies has strongly spurred the RESG approach. Reference [24] presents findings from an extensive literature survey uncovering existing network topologies that can be applied for combining IoT with Serious Games. Reference [25] presents the implementation, validation and experimental results of research aiming to apply physical rehabilitation monitoring combining Virtual Reality SGs and a Wearable Sensor Network to improve the patient engagement during physical rehabilitation. Reference [26] proposes the use of a new data toolchain for serious games analytics. The toolchain relies on the open source Measurify IoT framework [27], and takes advantage of its edge computing extension (namely, Edgine), which can be seamlessly deployed cross-platform on embedded devices and PCs as well, thus allowing adding intelligence directly to the sensors. The implementation on Unity 3D of a Virtual Reality use case is also presented there, showing the flexibility of IoT solutions that thus may also be applied, for instance, to state of the art driving simulators, such as Carla [28], AirSim [29] and GTA V [30].

Serious games that involve driving and mobility have been designed to support a range of objectives in intelligent transport systems, such as encouraging the use of different transport modes and supporting route planning [31]. Gamification solutions have been applied towards more fuel-efficient driving (e.g., [32]) and safer driver behavior (e.g., [33]). Social networking has been exploited as well. For instance, [34] developed an incentive system for comparing a driver’s FC average with other drivers in similar conditions. A social awareness system was presented to promote eco-driving and safe driving by analyzing driving patterns that are detected through the Global navigation satellite system (GNSS) and motions sensors [35]. Reference [14] implemented a gamified tool aimed at promoting fuel-saving based on a telemetry data comparison from similar vehicles. Drivers could share their scores with others, e.g., via social networks. Experimental results showed the effectiveness of gamification and eco-driving assistants for fuel-saving.

Car manufacturers are using gameful designs [36]—with a strategy based on the use of color and contrast that helps reduce glance time—to supply the drivers with virtual rewards which are presented with simple gaming interfaces (e.g., trees, flower or medals) based on their eco-driving achievements. For example, for the hybrid models in Ford’s ‘SmartGuage’ with ‘Ecoguide’, there is a game-changing instrument panel with a rich-color LCD screen with non-distracting animation that customizes real-time feedback about driver habits including fuel and battery power levels and average miles-per-gallon [37]. Another example is the Honda’s ‘Eco Assist’ (available on Insight, Civic, CR-V and Accord), featuring a feedback system for coaching drivers on fuel-efficiency. The cockpit integrates an interactive display providing a plant metaphor, where the number of leaves shown is an indicator of the current level of green driving [38]. To the best of our knowledge, the technological details of the manufacturer-specific solutions are not disclosed in the literature.

## 3. Experimental Settings

In order to conduct our feasibility investigation, we set up an experimental environment in a lab, utilizing data recorded in naturalistic driving. Since we think that the data workflow that we propose to game designers should be independent of original equipment manufacturer (OEM) proprietary solutions, we decided to extract vehicular signals through the on-board diagnostic (OBD-II) standard interface, which exposes diagnostic information circulating on vehicular buses [39]. This should allow developers to efficiently create games for different kinds of vehicles, without having to deal with the different OEM proprietary signal formats.

Thus, for the training and testing of the ML models, we used data from a naturalistic driving dataset extracted from enviroCar, a citizen science platform for collecting pseudonymized information from ordinary drivers in several European countries [40]. EnviroCar data are collected through standard Bluetooth OBD-II adapters to the vehicle’s Controller Area Network (CAN) bus. An Android smartphone app samples the signals (typically at a 5s sampling rate) and delivers the samples to the enviroCar server, together with GNSS information. Green drive factors (e.g., FC and Carbon dioxide (CO2) emissions) are calculated later on the cloud server.

**Figure 2:**
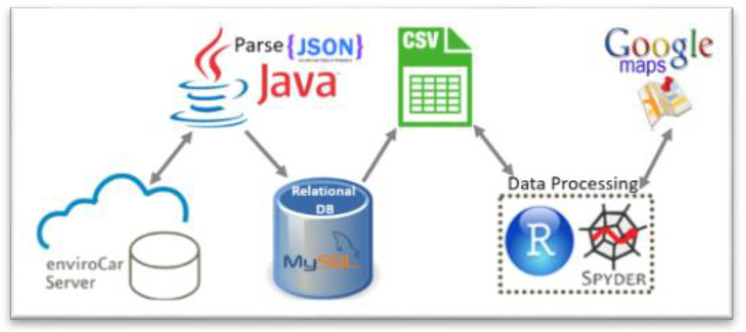
High-level scheme of the data preparation system architecture.

To set up our experimental environment, we designed and implemented a system architecture that obtains data from the enviroCar server through HTTP requests in Representational state transfer (REST) APIs and stores it in a local relational database (Figure 2). We exploited the Google Maps API provided by the “ggmap” R library to implement the back reverse coding [41], which translates each point’s location (GNSS latitude and longitude) into a readable address (country, locality and route). Our analysis considered 8726 gasoline tracks for a total of 983,291 measurements that were recorded mostly in Germany in the period 1 January 2012–15 June 2016. We focused on gasoline engines, since the estimation of FC (measured in liters/h) by enviroCar provides the best accuracy for these kinds of engines [40]. We worked with data recorded in different driving environments and not calibrated for a specific car model in order to target a certain degree of robustness.

## 4. Instant Fuel Consumption Estimation

The ‘engine fuel rate’ sensor is not mandatory in OBD-II, which leads to a need for estimating FC from parameters available from the OBD-II standard, independent of the car model [40].

EnviroCar estimates FC through Equation (1), which relies on the ‘air flow rate’ or ‘mass air flow’ (MAF) OBD-II sensor. MAF measures the amount of air that flows into the engine. It gauges the volume of air entering the vehicle’s fuel injection engine and sends this information to the engine control unit (ECU) to correctly balance the amount of fuel to the engine. Then, the ECU determines the amount of fuel that the injectors have to send to each one of the cylinders. Thus, the MAF sensor is a performance key of both the engine controller and the engine emission control system [42].

(1) $$\mathrm{Fuel}\;\mathrm{consumption}\left(\frac{g}{s}\right)=\frac{Fuel\;weight}{s}\left(\frac{g}{s}\right)=\frac{MAF\left(\frac{g}{s}\right)}{\mathrm{AFR}}$$

(2) $$\mathrm{Fuel}\;\mathrm{consumption}\left(\frac{l}{h}\right)=\frac{\frac{MAF\left(\frac{g}{s}\right)}{\mathrm{AFR}}}{Density\;of\;fuel\;\left(\frac{g}{l}\right)}\left(\frac{l}{s}\right)\times 3600\left(\frac{l}{h}\right)$$

MAF is measured in (grams/second). The mass air to fuel ratio (AFR) is 14.7 for gasoline. FC is given by the ratio between MAF and AFR, expressed in grams/second. Drivers are however more familiar with measuring FC in liters/hour, as in Equation (2), with the gasoline density being of 745 (g/L). Complete combustion involves a ratio of 14.7 kg of air per kg of gasoline. 

The MAF sensor is mandatory in the OBD-II standard; however, it is missing in some vehicle types. For this reason, the enviroCar community estimates its value by processing other measurements, such as temperature, air pressure and engine speed [40]. For the recommendation module, we were interested in exploring FC estimation using a different set of signals, particularly those that can be directly controlled by the driver, so to allow providing direct feedback for improving driving performance. Moreover, our predictor signals needed to not be specific for a single engine type. 

### 4.1. Data Analysis and Predictors Selection 

**Figure 3:**
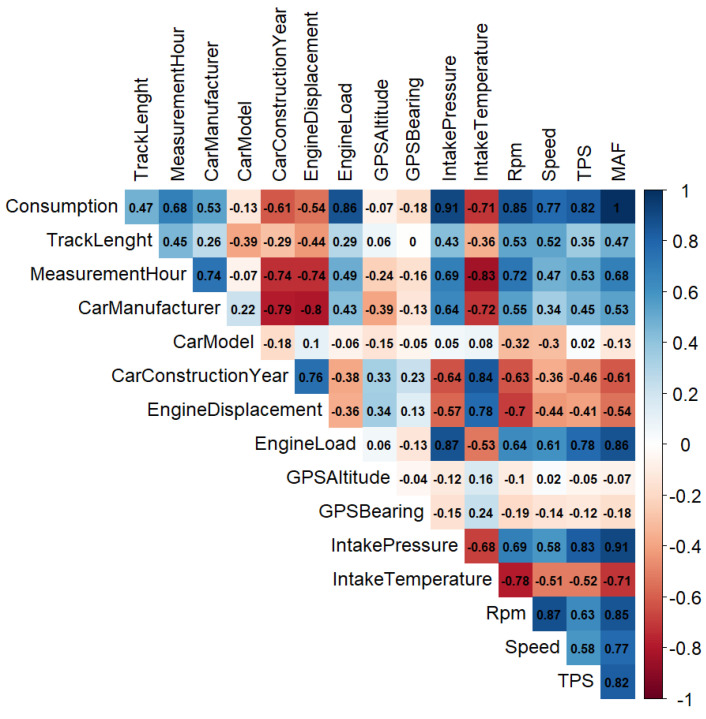
Correlation between enviroCar estimated fuel consumption and other variables (blue shades for positive correlation, red for negative).

As a first step, we analyzed the Pearson product-moment correlation (PPMC) between the target variable (estimated FC by enviroCar) and other variables (including vehicular signals), which is reported in Figure 3 (Table 1 explains the color codes). A positive sign indicates a positive correlation (vice-versa for a negative sign). Apart from MAF (with an obvious PPMC of 1), several signals are highly positively correlated with FC such as engine intake air pressure (0.91), engine load (0.86), RPM (0.85), TPS (0.82) and car speed (0.77). Intake-air temperature (−0.71) and car construction year (−0.61), on the other hand, are highly negatively correlated with FC.

We studied 14 predictors that might affect FC, as reported in Figure 3. They can be divided into four groups (Table 2).

### 4.2. Fuel Consumption Modeling 

We used the above-presented data to develop a new FC model based on ML. We applied three popular ML techniques: support vector machine for regression (SVR), random forest (RF) and artificial neural networks (ANNs), which we briefly introduce below.

The developed models were trained on 80% of the available data (786,633 samples) and tested on the remaining 196,659 samples to judge the quality of the fit. The models’ hyperparameter selection was performed by using 10-fold cross-validation to reduce overfitting. In particular, we relied on a grid search for tuning both SVR and RF models, while we used the random search to configure the ANN model. These hyperparameter optimization techniques were implemented using ‘GridSearchCV’ and ‘RandomizedSearchCV’, respectively, using the python ‘sk-learn’ library [45]. The grid search constructs and evaluates one model for each possible combination of the set of values defined by the user. The random search, instead, trains and assesses candidate models by using random combinations of the parameters for some iterations pre-set by an analyst.

In the following text, we describe the techniques used and their selected hyperparameters’ values.

*Support vector machine for regression (SVR)*

SVR represents the support vector machine (SVM) for regression problems. SVM [46] implements the principle of structural (instead of empirical) risk minimization, with valuable results for small training sets [47]. By using a “kernel trick”, an SVM can map a nonlinear problem into a linear one in a higher-dimensional space, allowing effective modeling of complex functional correlations [48]. The ‘SVR’ function of the ‘sklearn.svm’ python package was used to implement the SVR model. We used the common radial basis function (RBF) kernel SVR (or Gaussian kernel), as in [49,50]. Our cross-validation grid search spotted the following hyperparameter values: 1 for ‘C’ (which trades correct classification of training examples for maximization of the decision function’s margin) and 0.001 for ‘gamma’ (which defines the influence of a single training sample).

*Random forest (RF)*

RF is a popular ‘ensemble learning’ technique that generates a team of ML models to aggregate their results through majority voting (for classification) or by averaging (for regression) [51]. This strategy allows RF (that generates several different decision/regression trees) to be robust against overfitting and deliver a good performance [52]. We employed the sk-learn ‘RandomForestRegressor’, with a 10-fold cross-validation scheme to tune two key hyperparameters, such as the number of forest trees and the number of features to consider at each split. The grid search exploration shows that 800 trees with 9 features is an optimal configuration.

*Artificial Neural Networks (ANNs)*

ANNs are a popular tool to learn complex data patterns [53]. The ANN structure supports parallelization and adaptive learning to solve problems with collective processing, self-organization and fault tolerance. We implemented the ANN model by the means of the “Keras” python package [54], using a sequential model. Our random search cross-validation suggests that:

400 epochs are adequate for the entire training dataset, with 20 samples as inputs to the network before updating the weights (‘batch_size’). 

**Figure 4:**
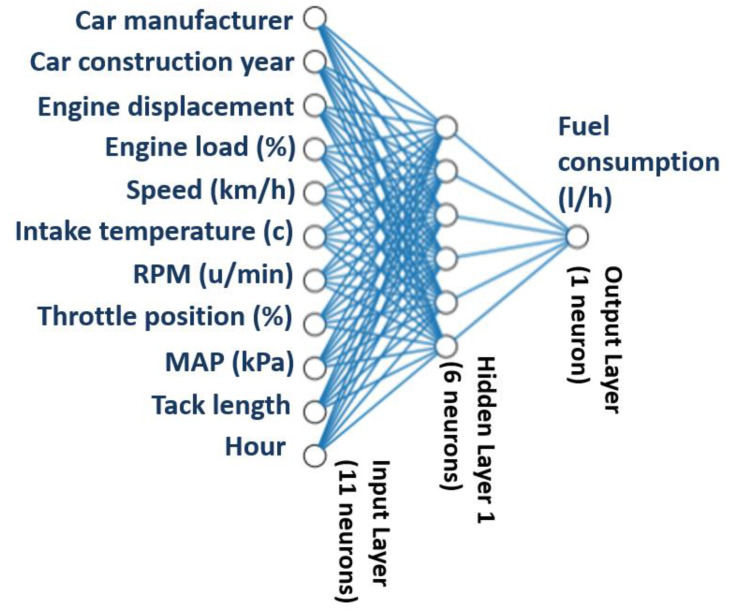
Representation of the ANN model developed.

A single hidden layer having 6 neurons provides satisfactory prediction accuracy. So, our ANN model’s structure consisted of one input layer (receiving data from the eleven inputs), one hidden layer (with six neurons) and one output layer (with one neuron for FC), as illustrated in Figure 4.

The ‘relu’ neuron activation function was used in all the hidden and output layers. ‘Adam’ was used as the optimization algorithm, with ‘normal’ for the NN weight initialization. 

No regularization was needed (‘dropout_rate’ = 0). The dropout regularization technique prevented complex co-adaptations on training data to limit overfitting.

## 5. Instant Driving Recommendations

The previous section targeted the real-time assessment of driver FC performance. Besides this, it is important to also provide a driver with guidance on how to improve their FC performance. Fuzzy logic (FL) is used in the development of human-like capabilities for artificial intelligence. It has the ability to deal with incomplete information [55] and to distinguish between different performance factors by matching any set of input-output data based on the estimated degree of truth. FL allows transferring human knowledge and expertise into a mathematical model consisting of if-then rules [56] that allows verbal feedback to be provided, directly related to the inputs. In our opinion, these features are also particularly well-suited for providing feedback to non-expert users. We thus built a FL model to give coaching advice to drivers, relying on just three OBD-II signals (namely, TPS, speed and RPM) that affect the consumption of fuel. Other factors (e.g., road-type, traffic conditions) do influence FC in a trip. However, for simplicity of data collection, we focused our research on these three variables because they are well known to and are directly controllable by the driver [57]. 

We studied all the possible combinations of the three chosen inputs, resulting in four fuzzy inference systems (FIS): FIS1 (TPS and RPM), FIS2 (speed and RPM), FIS3 (TPS and speed) and FIS4 (using all three inputs). We empirically defined the models’ membership functions and the fuzzy rules relying on a literature review and data analysis. Table 3 presents the driver feedback corresponding to the seven fuzzy rules that we deduced in case FC is a high ‘H’ or very high ‘VH’ after studying the 60 possible combinations of the membership functions of the variables with the AND operator, as detailed in [57]. The resulting runtime latency was always within 10 milliseconds.

While providing the recommendations, RPM is considered as the strongest FC predictor, followed by TPS and car speed. We deduced this feature importance rank while observing the fuzzy rules in [57] and then we confirmed it later through the RF feature importance interpretation tool for measuring the prediction strength of each variable in [58]. For instance, based upon the 4th rule in Table 3, if a ‘High’ FC is obtained because of a ‘High’ RPM, a ‘Medium’ TPS and a ‘High’ or a ‘Very high’ speed, then the system provides the advice “shift up the gear (and reduce speed)” prioritizing the gear shift action.

For providing verbal feedback, we also included an over-speed event detector [59]. Over speeding events are triggered if the vehicle’s speed overcomes the legal speed limit, which is obtained through a web service based on OpenStreetMap (OSM) [60]. Relevant feedback is provided to the driver based on thresholds.

## 6. Results/Case Study and Discussion

**Figure 5:**
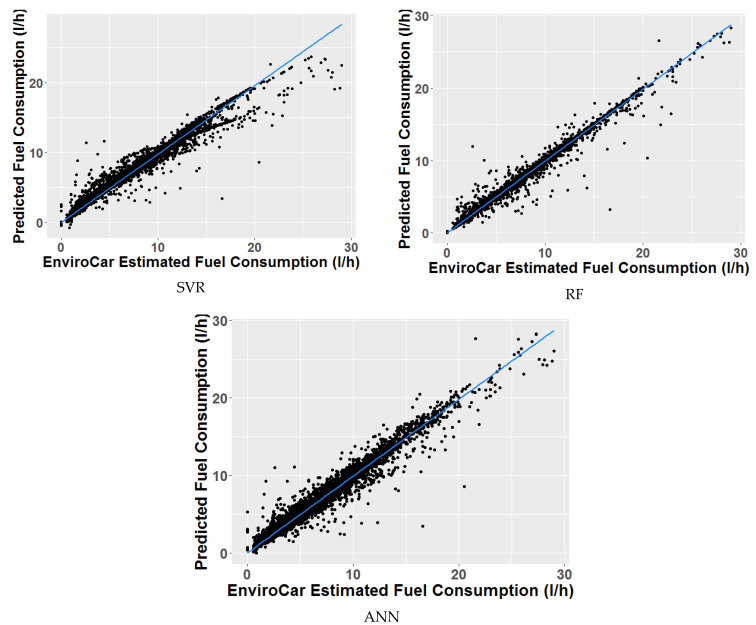
Comparison of the models’ correlations.

This section reports the results from experimental tests that were performed on an 8 GB RAM and i7-8550U CPU desktop computer. The out-of-sample performance of each ML model is expressed in terms of the most common statistical metrics for regression problems, such as mean-squared-error (MSE) and squared correlation coefficient (R2). Figure 5 illustrates the correlations of the three studied ML models. While Table 4 compares their performance metrices.

**Figure 6:**
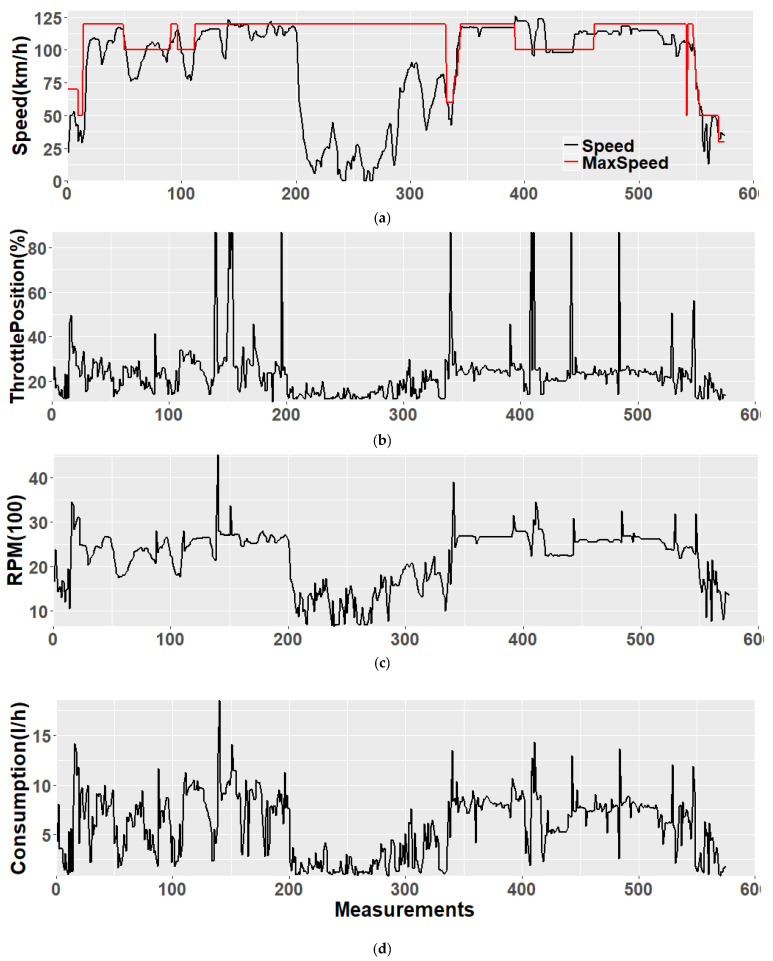
Performance for an example trip. Speed (also compared with the OSM speed limit in red) (**a**), throttle position (**b**), RPM (**c**) and fuel consumption (**d**). For all the sub-figures, the horizontal unit is the ordinal sample (measurement) number where the Sampling interval is 5 s.

Considering the driving recommendations, Figure 6 shows the time series of each one of the input signals (car speed, TPS and RPM in (a), (b) and (c), respectively) in one of the enviroCar tracks. FC is reported as well, in (d). The sample trip consists of 575 measurements, taken over about 50 min along a 71 km track. The trip was recorded in Germany on 17 February 2016, between 16:00 and 17:00, driving a Volkswagen Polo 9N 2009, gasoline engine. The trip was mostly driven on a highway, apart from two short initial and final segments. The speed is usually around 100 km/h, apart from the middle of the trace where we postulated that there could have been a traffic jam. The legal speed limit (provided by OSM and reported in red color) is respected for about 85% of the time (Figure 6a). There are few significant acceleration/deceleration spikes during the travel.

**Figure 7:**
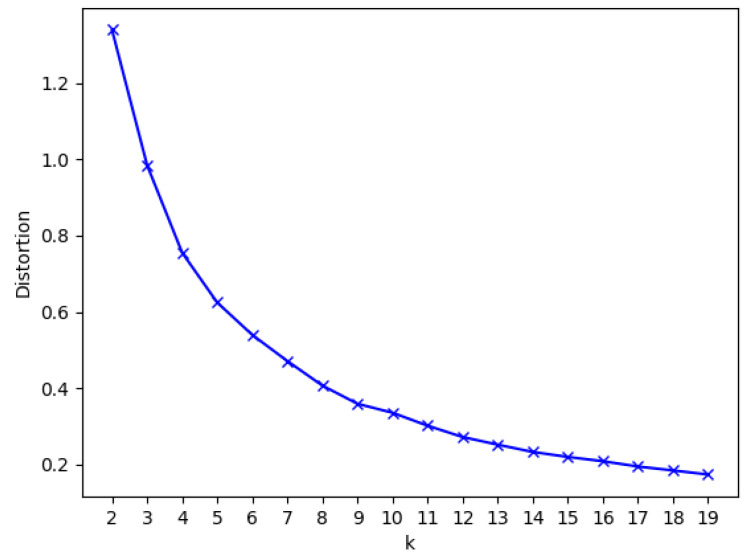
Elbow chart, giving a hint for defining the optimal number of driving style categories.

In eco-driving, important feedback to the driver is given by the driving style categorization, which is typically provided through the three traffic light colors (e.g., [61]). In order to have a quantitative basis for defining the best number of driving style categories, we relied on the “Elbow” method based on the 1d k-means algorithm. The elbow chart depicted in Figure 7 shows that the elbow (i.e., the point of inflection of the curve) is clearly above 3 (corresponding to the three traffic light colors) in our dataset. While 9 categories may be too many to communicate to the driver, 5 could be a decent trade-off. The corresponding cluster centroids are reported in Table 5.

For eco-driving profiling, a key metric is given by fuel-efficiency, which relates the amount of consumed fuel by a vehicle and the distance traveled, as in Equation (3). We estimated the fuel efficiency for a driver on a trip by dividing the estimated FC by the covered distance. Manufacturers provide a fuel efficiency figure for new cars in liters per 100 km under ideal conditions for urban, extra-urban and a mixture of the two, which is difficult to achieve. Hence, in order to allow comparisons, we normalized the values by the highest achieved fuel-efficiency with a similar car model, as in [62]. This avoids figuring out the maximum fuel-efficiency for every car model.

(3) $$\mathrm{Fuel}\_\mathrm{efficiency}=\frac{\mathrm{kilometres}\;\mathrm{traveled}}{\mathrm{total}\;\mathrm{trip}\;\mathrm{fuel}\;\mathrm{consumed}}$$

**Figure 8:**
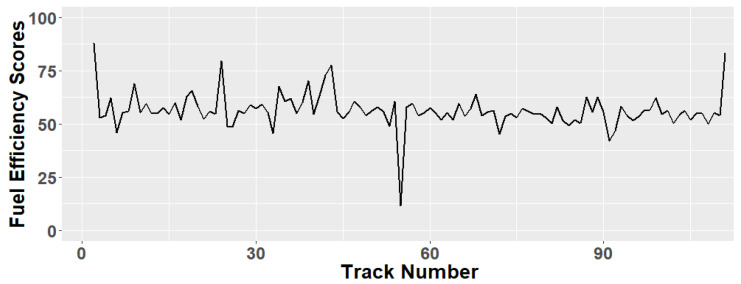
Fuel efficiency scores for 111 tracks for car model “Volkswagen Polo 9N 2009”, gasoline engine.

In our sample trip, fuel-efficiency was 0.021 km/L/h, while the maximum fuel efficiency (for the same car type, in the same area, in 111 trips) is 0.037 km/L/h. Figure 8 depicts the fuel efficiency scores (normalized to 100) for the 111 trips relevant to the same car model for the example journeys (Volkswagen Polo 9N 2009, gasoline engine). It can be noticed that it is common to attain a fuel efficiency score between 50 and 60.

**Figure 9:**
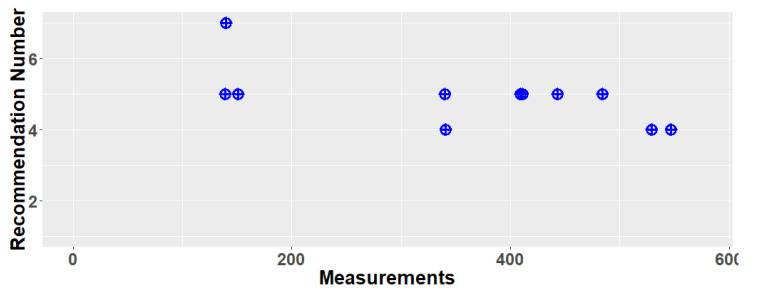
Driving recommendation timeline for the studied trip respecting Table 3′s seven rules.

Considering the instant verbal feedback, Figure 9 illustrates the timeline of the messages provided to the driver along the studied track, where each blue point represents a recommendation corresponding to one of the seven fuzzy rules reported in Table 3. We can see that rules number 5 and 4 are triggered quite frequently, while the others are much rarer. Looking at the structure of the rules, this particularly stresses the importance of properly tuning the gear shift.

Finally, as an example of the logical link between quantitative FC estimation (based on RF) and FL reasoning, we can consider the FC peak at sample number 140 in Figure 6. The actual values, reported in Table 6, show the triggering of the instant driving recommendation number 7. In particular, according to the FL membership functions, RPM is very high (VH) and TPS is high (H), and the estimated consumption of fuel is VH, which triggers the rule 7 message: “Shift up the gear”.

## 7. Conclusions and Future Work

IoT is spurring new types of typologies of serious games, such as RESGs, that support training directly in the field. In this context, a novel contribution of this work involves identifying two main features of RESGs (e.g., instant assessment and recommendation) and proposing a reference model featuring two modules employable in third-party RESGs to promote fuel-efficient driving (Figure 1). While this paper studied the specific case of automotive driving, the proposed model is general, and is thus applicable to other application domains such as health and fitness, as mentioned in the introduction, which can be followed up in future research. In fact, the two streams in Figure 1 are generic and could be exploited by different types of RESGs for training in a domain-independent way. Promising areas could be driving/piloting (with different types of transportation/logistic means), energy management or health/fitness, in real and/or virtual settings.

This paper also shows that machine learning algorithms can provide a valid implementation for such modules. As the first module estimates FC in real-time, we compared the performance of three well-established shallow machine learning algorithms: support vector machine for regression (SVR), random forest (RF) and artificial neural networks (ANNs). In our experiments, using the enviroCar public dataset, the algorithms show similar performance. RF achieves the highest correlation with FC. However, it takes the longest time for inference (27 ms, which is still suitable for a real-time requirement, provided that the further game processing chain up to the graphical user interface does not add a further significant delay). ANNs and SVR deliver a slightly worse performance, but with latency times within one millisecond.

The second module provides instant recommendations, suggesting actions to be taken when an inefficient driving pattern has been detected. This module can be effectively implemented using fuzzy logic. Also in this case, the latency (10 ms) is suitable for the real-time messaging requirement.

The algorithms exploit signals available through the OBD-II standard interface, which is a standard part of vehicles and is easily usable for IoT applications. We processed enviroCar data recorded from different car models, with the same engine and in various driving environments.

While we performed extensive lab tests, real-world tests with vehicular SGs are needed to verify the training and coaching validity of our approach. It will be interesting to see how game designers will creatively exploit this information while compelling SGs for promoting fuel efficiency. The work should be extended by also considering other types of vehicle engines. Moreover, other ML algorithms could be studied, such as reinforcement learning for providing recommendations. Another possible investigation concerns the use of smartphone sensors, thus avoiding OBD-II adapters, and the addition of other environmental factors, such as traffic and road types. FC is an important dimension of driving, but not the only one. Further research could take into account and assess other aspects, particularly safety. We believe that the proposed modules are also relevant due to the increasing levels of driving automation.

A key word of caution is finally needed concerning users’ privacy, as the proposed modules rely on highly sensitive data, and provide information that should not be misused nor shared [63,64,65].

## Figures and Tables

**Table 1:** Color code for the Pearson’s Correlation Coefficient.

Event Detector	Value	Classification
Small	0.1 to 0.3, light blue	−0.1 to −0.3, light coral (shade of orange)
Medium	0.3 to 0.5, mid blue	−0.3 to −0.5, mid coral
Large	0.5 to 1, dark blue	−0.5 to −1, dark red
No correlation	0, white	0, white

**Table 2:** Fuel consumption predictors and their characterization.

Predictor Group	Variables	Notes
Related to car characteristics	Car manufacturer; car model; car construction year; engine displacement	-
Read from the car’s internal sensors (OBD-II scanner)	Engine load, “%”	Indicates the amount of air and fuel being sucked into the engine
	Speed, “km/h”	The actual speed of the vehicle shown by the odometer (when there is no readable value from the speed sensor, we rely on the GNSS derived speed): speed burns fuel
	Intake-air temperature, “c”	Senses the air temperature inside the cylinders into the engine. This information is provided to the ECU for correcting the mixture formation and the ignition to determine the correct amount of fuel needed for optimum engine performance and economic outcomes
	Number of engine revolutions per minute (RPM)	Fuel consumption is typically related to high RPM [43]. Optimal RPM depends on the vehicle’s engine characteristics and on the road slope as well
	Throttle position sensor (TPS) or accelerator, “%”	Regulates the engine’s air and fuel intake. It is directly controlled by the driver, thus representing a fundamental element for comprehensible feedback and coaching
	MAF “L/s”	Presented above
	Intake manifold absolute pressure (MAP)	Used by the ECU to compute the MAF
Computed post-hoc and added on the community’s server	FC “L/h”	Described in Section 4
	Calculated MAF “g/s”	For the cases when the OBD adapter delivers no result for MAF [44]
	Track’s length	Track traveled distances in kilometers
Embedded sensors in Smartphone and timestamp data	GNSS speed	(When OBD-II adapter delivers no result for speed, the GNSS speed value is considered in this work) and the time of day (in hours)

**Table 3:** Fuzzy logic rules and corresponding feedback for high and very high FC levels (L: Low, M: Medium; H: High, VH: Very High).

Indicators			Estimation	Driver’s Feedback
RPM	TPS	Speed	FC	
L	H	H	VH	Shift down the gear (and raise the accelerator pedal)
L	H	VH	H	Shift down the gear and raise the accelerator pedal
H	M	H or VH	H	Shift up the gear (and reduce speed)
H	M	M	H	Shift up the gear
H	H		H	Shift up the gear (and raise the accelerator pedal)
VH	M		H	Shift up the gear
VH	H		VH	Shift up the gear

**Table 4:** Comparison of the Models’ Performance.

Performance Metric	SVR	RF	ANN
MSE	0.06	0.02	0.05
R^2^	0.98	0.99	0.98
Training time (min)	154	12	117
Inference time (ms)	1	27	0.28

**Table 5:** Centroids of the Clusters for K = 5 and K = 9.

k	Centroids (L/h)
5	1.76, 4.35, 6.93, 9.04, 11.91
9	1.37, 2.65, 4.15, 5.68, 7.17, 8.46, 9.73, 11.45, 14.84

**Table 6:** Driving feedback for a sample of the studied track (H: high, VH: very high).

Event Detector	Value	Classification
RPM	4530	VH
Speed/OSM speed	117.36/120 km/h	H, respect the speed limit
TPS	87%	H
**Driving Recommendation**		
“Shift-up the gear”		

## Data Availability

The raw data analysed in this study is available from https://envirocar.org/resources.html?lng=en, accessed on 19 May 2021.
